# A Data-Driven Evaluation of the Stop TB Global Partnership Strategy of Targeting Key Populations at Greater Risk for Tuberculosis

**DOI:** 10.1371/journal.pone.0163083

**Published:** 2016-10-12

**Authors:** Zoë M. McLaren, Kathryn Schnippel, Alana Sharp

**Affiliations:** 1 Department of Health Management and Policy, School of Public Health, University of Michigan, Ann Arbor, Michigan, United States of America; 2 Research Department, Right to Care, Centurion, South Africa; 3 Clinical HIV Research Unit, Department of Internal Medicine, School of Clinical Medicine, Faculty of Health Sciences, University of the Witwatersrand, Johannesburg, South Africa; Agencia de Salut Publica de Barcelona, SPAIN

## Abstract

**Objective:**

Identifying those infected with tuberculosis (TB) is an important component of any strategy for reducing TB transmission and population prevalence. The Stop TB Global Partnership recently launched an initiative with a focus on key populations at greater risk for TB infection or poor clinical outcomes, due to housing and working conditions, incarceration, low household income, malnutrition, co-morbidities, exposure to tobacco and silica dust, or barriers to accessing medical care. To achieve operational targets, the global health community needs effective, low cost, and large-scale strategies for identifying key populations. Using South Africa as a test case, we assess the feasibility and effectiveness of targeting active case finding to populations with TB risk factors identified from regularly collected sources of data. Our approach is applicable to all countries with TB testing and census data. It allows countries to tailor their outreach activities to the particular risk factors of greatest significance in their national context.

**Methods:**

We use a national database of TB test results to estimate municipality-level TB infection prevalence, and link it to Census data to measure population risk factors for TB including rates of urban households, informal settlements, household income, unemployment, and mobile phone ownership. To examine the relationship between TB prevalence and risk factors, we perform linear regression analysis and plot the set of population characteristics against TB prevalence and TB testing rate by municipality. We overlay lines of best fit and smoothed curves of best fit from locally weighted scatter plot smoothing.

**Findings:**

Higher TB prevalence is statistically significantly associated with more urban municipalities (slope coefficient *β*_*1*_ = 0.129, *p* < 0.0001, *R*^*2*^ = 0.133), lower mobile phone access (*β*_*1*_ = -0.053, *p* < 0.001, *R*^*2*^ = 0.089), lower unemployment rates (*β*_*1*_ = -0.020, *p* = 0.003, *R*^*2*^ = 0.048), and a lower proportion of low-income households (*β*_*1*_ = -0.048, *p* < 0.0001, *R*^*2*^ = 0.084). Municipalities with more low-income households also have marginally higher TB testing rates, however, this association is not statistically significant (*β*_*1*_ = -0.025, *p* = 0.676, *R*^*2*^ = 0.001). There is no relationship between TB prevalence and the proportion of informal settlement households (*β*_*1*_ = 0.021, *p* = 0.136, *R*^*2*^ = 0.014).

**Conclusions:**

These analyses reveal that the set of characteristics identified by the Global Plan as defining key populations do not adequately predict populations with high TB burden. For example, we find that higher TB prevalence is correlated with more urbanized municipalities but not with informal settlements. We highlight several factors that are counter-intuitively those most associated with high TB burdens and which should therefore play a large role in any effective targeting strategy. Targeting active case finding to key populations at higher risk of infection or poor clinical outcomes may prove more cost effective than broad efforts. However, these results should increase caution in current targeting of active case finding interventions.

## Background

Tuberculosis is a significant global health threat and kills more people each year than any other infectious disease [1]. Since tuberculosis (TB) is a treatable and curable infection, identifying persons infected with TB is an important component of reducing transmission and population prevalence. In 2015, the Stop TB Global Partnership launched an initiative to end TB. According to the 5-year Global Plan 2015–2020, all countries should aim to diagnose and treat 90% of all persons with active TB, with a specific focus on ‘key populations’ at greater risk for infection or poor clinical outcomes [2]. Typically, these risk factors include housing and working conditions, incarceration, low household income, malnutrition, co-morbidities, exposure to tobacco and silica dust, barriers to accessing medical care, and stigma. Since the characteristics of key populations vary by context, the Global Plan encourages countries to identify key populations at the sub-national level and regularly report progress on operational targets disaggregated by key population group. To achieve this goal, the global health community needs effective, low cost, and large-scale strategies for identifying and targeting key populations.

One promising strategy is to use regularly-collected sources of data to identify key populations and develop granular estimates across entire countries at low cost. Using South Africa as a test case, we assessed the feasibility of such a strategy using a large dataset of TB tests from the public sector laboratory network in combination with census data to identify risk factors for TB infection. We analyzed characteristics of local municipalities, as reported in census data, to assess how sub-national data can accurately inform the targeting of key populations in need of active TB case finding.

This strategy is applicable to any other country with access to TB testing and census data, and allows countries to tailor their outreach activities to the particular risk factors of greatest significance in their national context. Since laboratory registry data and census data are regularly collected in many low and middle income countries, using these forms of electronic data to guide active case finding strategies may significantly improve the effectiveness of these outreach activities at a lower cost than more general case finding programs, thereby improving individual outcomes, minimizing transmission, and reducing TB burden.

The annual TB incidence rate in South Africa is the second highest in the world (estimated to be 834/100,000 population) and TB has remained the leading cause of death for the past decade [1,3]. South Africa has not yet completed a TB prevalence survey; incidence is estimated by the WHO at the national level using transmission modeling. In 2015, the South African Department of Health with support from UNAIDS and the South African National AIDS Council completed its first ever TB Investment Case, which used the TIME Impact model within the Spectrum modeling suite to evaluate the impact of additional investment in TB control [4,5]. As a result of the TB Investment Case, the South African Minister of Health announced a five-year campaign to target key populations with active TB case finding with the aim of achieving the Global Plan targets [6]. The national campaign identified high-risk populations as those living in informal settlements or poorly ventilated households, people who are malnourished, migrant populations, miners, incarcerated persons, young children, and those living in six priority districts with a high density of mines.

## Methods

We collected data from two large datasets to identify correlations between TB prevalence and risk factors. We used data from the National Health Laboratory Service (NHLS) database of TB diagnostic tests performed on patients in all South African public health facilities to estimate TB infection prevalence at the municipality level. Using data from the 2011 South African census, we estimated municipality rates of urban households, informal settlements, household income, unemployment, and cell phone ownership. By merging these sources of data, we were able to estimate the utility of these demographic features in locating key populations.

### NHLS data

We collected TB testing data from all patients aged 16–64 tested in South African public health facilities in 2010 for a total of 1,194,122 tests. We defined TB prevalence as the number of patients with at least one TB positive test as a proportion of the census population. Coverage of TB case finding is defined as the number of patients tested for TB by either smear microscopy, polymerase chain reaction (PCR), or culture test as a proportion of the census population. Patients with multiple episodes of TB in 2010 are counted as a single case.

### Census data

Data on demographic features are obtained from the 2011 South African census. We estimate municipality population rates of urban households, informal settlements, household income, unemployment, and cell phone ownership. Definitions of the variables used in these analyses are described by Statistics South Africa [7]. The census reports annual household income in twelve categories; we grouped the lowest three categories to create an indicator for families earning less than 9,600 South African Rand per year (equal to approximately $1,430 in 2010).

### Analysis

We plot the set of population characteristics against TB prevalence and TB testing rate by municipality overlaid with lines of best fit and smoothed curves of best fit from locally weighted scatter plot smoothing. We perform linear regression analysis with the formula *Y = β*_*0*_ *+ β*_*1*_*X + ε*, where *Y* represents either TB prevalence or TB testing rate and *X* represents municipality rates of demographic characteristic from the census data (urban households, informal settlements, household income, unemployment, and cell phone ownership). Regression coefficients (*β*_*1*_) and coefficients of determination (*R*^*2*^) are reported.

### Data limitations

Data from seven municipalities in the Eastern Cape was not included in the NHLS data (Blue Crane Route, Gariep, Inkwanca, Ngqushwa, Nxuba, Port St. Johns, and Tsolwana) and they were therefore excluded. One outlier municipality (Cederberg) was excluded from the analysis. In the year analyzed (2010), the province of KwaZulu Natal was not part of the national laboratory network and therefore municipalities in KwaZulu Natal were excluded from the analysis due to the unavailability of TB test data.

### Ethics approval

Ethics approval was obtained from the University of Michigan Institutional Review Board, Ann Arbor, MI, USA, and the University of Cape Town Faculty Ethics in Research Committee, Cape Town, South Africa. As this study was a secondary analysis of routinely collected anonymized data, written consent was not required.

## Results

### Urban households

**Fig 1:**
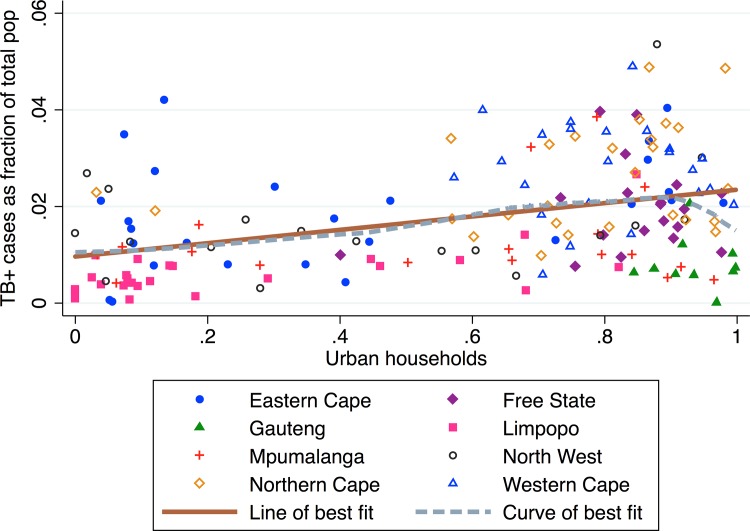
TB prevalence vs. proportion urban households by municipality.

Fig 1 shows the relationship between the municipality proportion of households categorized as urban and municipality TB burden. In our data, we observe that municipalities with a higher proportion urban households have, on average, higher TB prevalence. There is a significant positive association (*β*_*1*_ = 0.129, *p* < 0.0001, *R*^*2*^ = 0.133) between the proportion of urban households and TB prevalence in a municipality. There are several outlier largely urban municipalities in Gauteng province with low TB prevalence, as well as several rural outlier municipalities in the Eastern Cape province with high TB prevalence.

### Informal settlements

**Fig 2:**
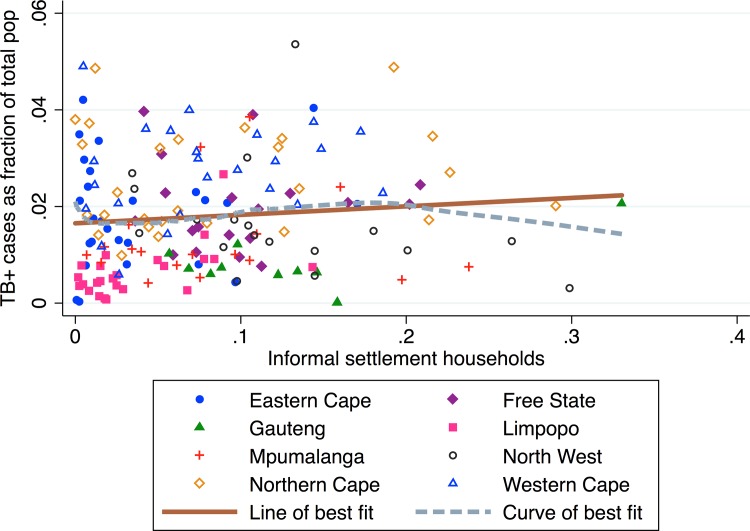
TB prevalence vs. proportion households in informal settlements by municipality.

We plot the proportion of households that are informal settlements and TB prevalence, by municipality, in Fig 2. Overall, we observe that most municipalities have a low proportion of informal settlement households (mean of 7.6%). We do not observe a significant linear association between the informal settlement proportion and the TB prevalence (*β*_*1*_ = 0.021, *p* = 0.136, *R*^*2*^ = 0.014). In the municipalities with a high proportion of informal settlement households, there is a negative association between informal household and TB prevalence; however, this relationship is primarily driven by Madibeng, a large outlier municipality with a high density of platinum mines, a high informal settlement proportion, and low TB prevalence.

### Mobile phone access

**Fig 3:**
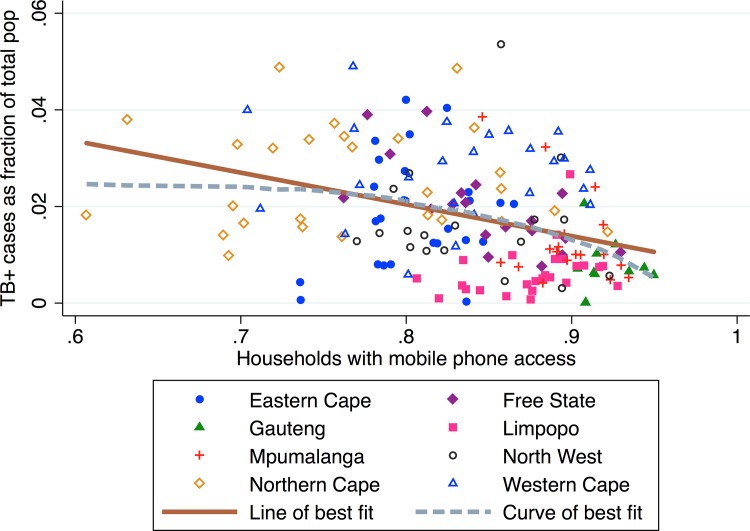
TB prevalence vs. proportion households with access to mobile phone by municipality.

In Fig 3, we plot TB prevalence against the proportion of households with access to a mobile phone. Access to mobile phones is high in South Africa, with an average of 89.5% of households in our sample having a working mobile phone in their homes. We find that municipalities with high mobile phone access have lower TB prevalence. There is a negative linear association between the proportion of households with access to a mobile phone and the TB prevalence (*β*_*1*_ = -0.053, *p* < 0.001, *R*^*2*^ = 0.089).

### Unemployment rate

**Fig 4:**
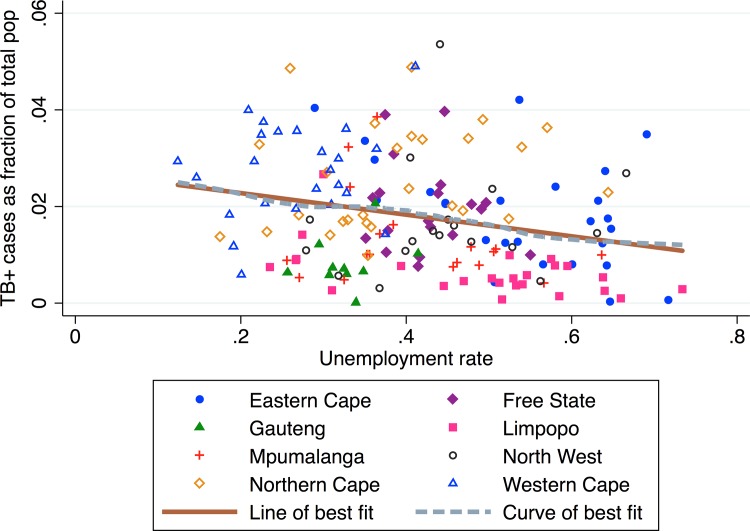
TB prevalence vs. unemployment rate by municipality.

Fig 4 shows the relationship between municipality unemployment rate and TB prevalence. We find that higher unemployment rates are associated with lower TB prevalence. There is a negative linear association between the unemployment rate and the TB prevalence (*β*_*1*_ = -0.020, *p* = 0.003, *R*^*2*^ = 0.048). By contrast, municipalities in Gauteng have low TB prevalence despite the low unemployment in these municipalities (mean municipality unemployment rate of 12.9%). We find a wide range of unemployment rates in the Northern Cape province and overall high TB prevalence.

### Household income

**Fig 5:**
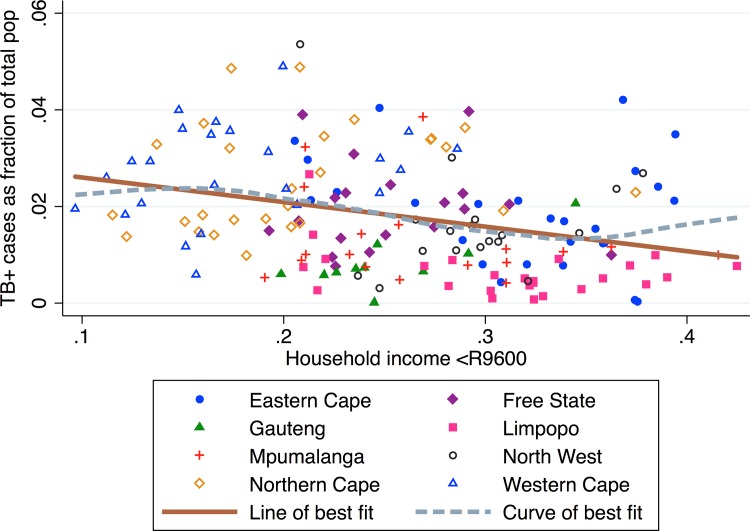
TB prevalence vs. proportion of households with very low annual income (less than 9,600 ZAR or 1,430 USD in 2010) by municipality.

We plot the proportion of households with earnings less than 9,600 South African Rand (equal to $1,430 in 2010) and TB prevalence (Fig 5). We find that municipalities with a higher proportion of low income households have, on average, lower TB prevalence. There is a negative correlation between low-income households and TB prevalence (*β*_*1*_ = -0.048, *p* < 0.0001, *R*^*2*^ = 0.084). The exceptions to this trend are municipalities in the Eastern Cape, which have high TB prevalence and a high proportion of households with low income.

**Fig 6:**
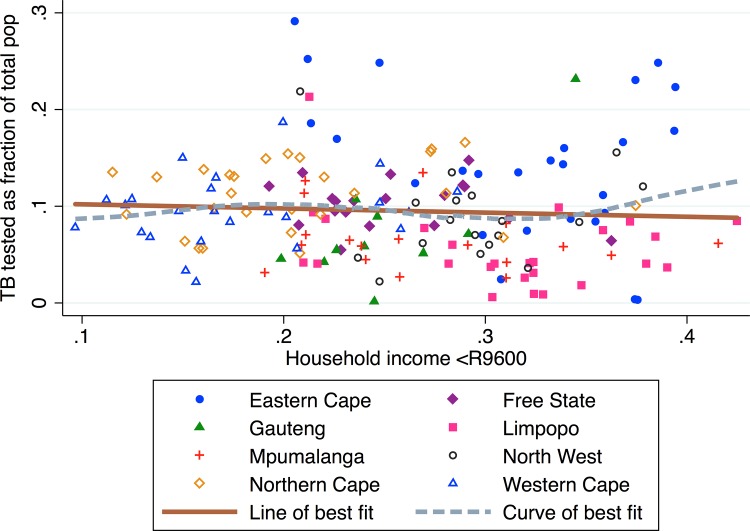
Number of patients tested for TB as a fraction of municipality population vs. proportion of households with very low annual income (less than 9,600 ZAR or 1,430 USD in 2010) by municipality.

To identify whether the negative association between low income households and TB prevalence is a by-product of higher case finding, we compare low income households with TB testing rates (Fig 6). We plot TB testing rates and households incomes and find that municipalities with more low income households have marginally higher TB testing rates. However, this association is not statistically significant (*β1* = -0.025, *p* = 0.676, *R*^*2*^ = 0.001), so municipality household income levels are not associated with TB testing rates. The curve of best fit reveals a positive correlation between low household income and TB testing in the low (>30% low household income) income municipalities, particularly in the Eastern Cape.

## Discussion

Reducing the TB burden will require both improved case finding and timely treatment. Passive case finding, long promoted by the WHO in developing countries as part of the DOTS strategy, has failed to control TB in South Africa [8]. Active case finding strategies range from surveying out-patients in hospitals to aggressively screening in communities. These strategies are more labor-intensive and expensive for the health system than passive case finding, but several modeling studies find that active case finding may produce significant reductions in TB incidence and mortality [9–11]. Targeting active case finding to key populations at higher risk of infection or poor clinical outcomes may prove more cost effective than broad efforts. This analysis focuses on population characteristics and healthcare factors that may guide case finding strategies for key populations. We highlight several factors identified by the Global Plan as those most associated with high TB burdens and which should therefore play a large role in any effective targeting strategy. We focus our analysis on informal settlements, urban communities, and poverty.

We find that a higher TB burden is correlated with more urbanized municipalities but not with informal settlements. Urban areas tend to have lower unemployment, since there are greater job opportunities in highly populated areas, but also contain urban informal settlements. An estimated 57% of South Africans living in urban environments reside in slum conditions with a high TB burden [12]. Greater employment is associated with lower poverty; however, employment itself may confer a greater risk of TB infection if employment is associated with an elevated risk of infection in mines and mining communities, due to both crowded living and working conditions and silicosis [13,14]. Employed populations may artificially appear to have higher TB prevalence due to improved case finding activities through employment-related programs, particularly among miners [15]. Similarly, informal settlements are less ventilated, crowded and allow for TB transmission (although this relationship is not statistically significant).

We analyze three demographic characteristics as proxies for poverty: household income, unemployment, and access to a mobile phone. We find that higher TB prevalence is correlated with lower mobile phone access, lower unemployment, and higher income. The finding that measures of higher income are associated with higher TB prevalence may be explained by lower TB testing rates in low income municipalities; however, we observe that TB testing is not associated with one measure of poverty (household income). Note that we analyze the proportion of households within a municipality that are low income but do not measure the size of the low income household population; as such, large municipalities with a large number, but small proportion, of households that are low income may mask the size of the low income population.

None of the sub-national graphs presented here show a clear association between these population factors and the TB burden. The three measures of poverty in our analysis (household income, unemployment, and cell phone access) have different directions of association with the TB burden. In our data, the strongest predictor of TB prevalence is the proportion urban households in a municipality. Overall, these analyses reveal that this set of population characteristics that define key populations do not adequately predict populations with a high TB burden.

One limitation of the analysis is that we do not have a “gold-standard” measure of population TB burden at local levels because South Africa has yet to complete a TB prevalence survey. For this analysis, we used laboratory reports of detected TB cases as a proxy for prevalence. Additionally, the exclusion of KwaZulu Natal province from the analysis due to data unavailability may have biased our findings since the province reports high TB rates. While we found no statistically significant association between the TB testing rate and the population characteristics examined, low rates of TB testing could lead to an under-reporting of the TB burden in the NHLS data. Poverty is a complex phenomenon and the variables we analyze, which focus on both income and asset measures, cannot fully capture the TB risk factors associated with poverty. However, our analysis demonstrates that the effectiveness of single-factor targeting is highly dependent on the choice of factor. Care must be taken to validate any data-driven targeting strategy.

Making progress against TB will require improvements in diagnostics and treatment, scaling up of prevention measures, and identifying new therapies and diagnostic technologies [16]. Identifying key populations at high risk of TB infection can allow for better targeting of interventions and resources and more cost effective health programs. The use of large and regularly collected datasets can identify these populations by analyzing population features and TB burden [17]. We piloted this method with a test case using data from the census and from the registry of TB tests. We find that the census demographic characteristics queried in our analysis do not reliably predict populations with higher TB prevalence, or ‘key populations’. National-level TB prevalence modeling cannot simply be adjusted to sub-national estimates on the basis of municipality population characteristics. These results should increase caution in the current targeting of active case finding interventions and improved TB screening more generally. Future work should identify factors that play a large role in any effective targeting strategy.
